# Measuring inequalities in the selected indicators of National Health Accounts from 2008 to 2016: evidence from Iran

**DOI:** 10.1186/s12962-020-00235-7

**Published:** 2020-09-22

**Authors:** Mohammad Hossein Mehrolhassani, Vahid Yazdi-Feyzabadi, Marzieh Lashkari

**Affiliations:** 1 Social Determinants of Health Research Center, Institute for Futures Studies in Health, Kerman University of Medical Sciences, Kerman, Iran; 2 Medical Informatics Research Center, Institute for Futures Studies in Health, Kerman University of Medical Sciences, Kerman, Iran; 3 Health Services Management Research Center, Institute for Futures Studies in Health, Kerman University of Medical Sciences, Kerman, Iran

**Keywords:** Inequality, Financing agents, National health accounts, Gini coefficient

## Abstract

**Background:**

Increase in total health expenditures is one of the main challenges of health systems worldwide, and its inequality is considered as a concern in global arena especially developing countries. This study aims to measure inequality in the distribution of selected indicators of national health accounts across the Iranian provinces.

**Methods:**

In this study, the data on health financing agents from provincial health accounts from 2008 to 2016 were collected. Gini coefficient (GC) was used to measure inequality. The population and the number of service providers in each province were the bases to measure the GC. The Coefficient of Variation (CV) and the Rate Ratio (RR) were used to determine the dispersion and variation across the provinces. Disparity index was employed to measure the average deviation of the out-of-pocket (OOP) proportion from the desired OOP proportion presented in national development plans (NDPs) of Iran.

**Results:**

The distribution of resources using both bases were unequal, especially in OOP, with the highest rate over the years studied, ranging from 0.50 to 0.59. The inequality in public resources was lower, with Health Insurance Organization dropping from 0.42 to 0.40 over the years. CV and RR also confirmed the inequality in health resources distribution. In the years 2014 and 2015, the lowest and highest levels were 0.22 and 0.39, respectively. The values of disparity index for OOP had a fluctuating trend ranging from 37.01 to 65.85%.

**Conclusion:**

Inequality in the distribution of public health expenditures was moderate to high. Moreover, inequality in private health expenditures was higher than public one. Distribution of OOP spent by households at provincial level showed a high inequality. It is suggested that inequality measures to be considered in NDPs to illustrate how resources are distributed at the geographical level. NHA framework can help to provide robust evidence base for policymaking.

## Background

Health provision and promotion are essential for the welfare as well as the social and economic development of societies [1, 2]. Consequently, most governments have considered health as a matter of governance to meet individuals’ expectations and have taken responsibility for the establishment and maintenance of the health system [3, 4]. The increase in public expectations and disease burden, development of new medical technologies, and limitation of the health system resources [5] hinder the access to affordable health care and raise equity in health financing as a global challenge, particularly for low- and middle-income countries [6, 7]. In order to improve community health and provide fair and need-based health services, participation, coordination, and control of all the public and non-governmental actors are one of the main tasks covered by health governance in each health system.

Health financing directly effects on universal health coverage (UHC), the goal declared in sustainable development goals, which seeks to utilization of quality health care for all, according to need, along with equitable financial contributions and protection against catastrophic health expenditure. Thus, it is essential to provide equitable financing mechanisms to ensure that all people have access to a wide variety of health services with adequate quality and being efficient; and that the usage of these services does not subject the consumer to financial hardship [7, 8]. To achieve these mechanisms requires applying appropriate and tailored laws and regulations and their implementation to regulate all actors involved in service provision, resource generation, pooling of financial resources, and the strategic purchasing of health services [4]. The financial resources performance with four sub-functions of revenue raising, pooling, purchasing, and benefits design is a crucial factor in access to and fair contribution in supplying resources [9, 10]. In this regard, one of the main concerns of policymakers is how financial resources agents are distributed to ensure people's fair contribution in financing across the geographical range [11, 12].

The contribution of public and private sectors, particularly the share of direct out-of-pocket (OOP) payments, are the key indicators of how to support the financial resources [8, 13]. The high proportion of OOP indicates that the performance of the financial resources regarding the risk spreading and pooling does not work correctly, resulting in inequity in financial contribution. This issue is especially crucial for high-risk and low-income groups in accessing health services. Furthermore, the public sector contribution is more dependent on the natural and oil resources or tax revenues that may vary from one country to another country; this justifies different levels of resource sustainability [14].

National health accounts (NHA) are recognized as a leading framework for collecting, compiling, and analyzing data on health expenditures to and within the health system. It describes where the money comes (private and public sectors) from and how it is spent and allows policymakers with adequate information on health resource flows. This tool also may provide the same information at provincial levels, and describe how health resources are collected, utilized, and spent. This information may help to provide appropriate financial evidence for better health financing decisions. One of the main sections in this tool is the information on how resources are distributed and allocated among different public and private actors in health financing, and health service spending and purchasing [8, 15, 16].

Total health expenditure (THE), OOP, and the share of public and private actors are among the main indicators attained from national health accounts in each country, which may also be calculated at the provincial level. To investigate how these resources are varied at the provincial level, various indicators such as the Gini coefficient (GC), the coefficient of variation (CV), and Theil and Atkinson indices are employed across the country provinces. One of the most common and widely used measures is the GC [17], investigating the trends provides some evidence for informed based policymaking to improve health financing policies.

Different studies have been conducted on measuring the indicators of equity in health financing across the world. Séne and Cissé reported that the type of providers, as well as geographical access to health providers, affect OOP spending by households [18]. In Iran, the results of a study conducted at the provincial level showed that there is a gap between OOP during the years studied with the goals set (OOP to be reduced to 30%) in the national development plans (NDPs) [19]. Another study conducted in Iran showed that catastrophic health expenditures (CHE) occurrence in rural areas was higher than in urban areas. Furthermore, some provinces such as Fars, East Azerbaijan, Markazi, Kerman, and Guilan had a CHE occurrence higher than less-developed provinces such as Sistan & Baluchestan [20].

Iran’s health system has experienced various reforms in the area of financing during past decades. One of the most significant reforms that have been recently implemented is the health transformation plan (HTP). The plan began in 2014 with a focus on increasing the contribution of the government and health insurers to health expenditures in provinces. It also applied some motivational policies to ensure fair and equitable distribution of physicians and subspecialists throughout the country. All these interventions were implemented in alignment with the decrease of the OOP share significantly [21]. Although some previous evidence showed a significant reduction in the OOP share [19], there is insufficient evidence of inequality in the distribution of OOP spending and health financing agents at provincial levels. Moreover, the fourth and fifth NDPs in Iran have emphasized on improving financial protection indicators such as reduce in OOP spending to 30%, and a decrease in the number of households facing CHE to about 1% [22, 23]. In summary, the present study aimed to measure the inequality in the geographic distribution of financial resources with a focus on the main indicators of provincial health accounts, indicating health financing agents in Iran.

### Health care financing system in Iran

The health system of Iran is organized at three levels: national, provincial, and district. At national and provincial levels, the MoH and Medical Sciences Universities (MSUs) are responsible for the health of the covered population. Furthermore, at the district level, the district health networks provide primary health care and basic medical services in a catchment area focus. The health care financing in Iran is a mixed financing system with different types of financing agents that function alongside each other [24]. On the basis of NHA categorization, the main health care financing agents in Iran are the public and private sectors. The public sector consists of governmental and non-governmental sectors. In this case, the governmental sector encompasses government budget allocated by MoH and MSUs at national and provincial levels, respectively. The non-governmental sector also includes four main public health insurance organizations that purchase inpatient and outpatient services from both public and private providers to some extent [25]. These health insurance organizations include Social Security Insurance Organization (SSIO), Iran Health Insurance Organization (HIO), the Armed Forces Medical Services Insurance Organization (AFMSIO), and Imam Khomeini Relief Foundation (IKRF) health insurance. Of these, SSIO and HIO cover over 90% of the insured population in Iran.

It should be noted that in addition to purchasing health care services, SSIO also directly delivers health services to the covered population through its outpatient and inpatient centers. According to a national demographic and health survey conducted in 2010, about 83% of the population benefits from a basic social health insurance coverage. It is estimated that the population coverage has been increased to over 95% following HTP implementation in early 2014 [26]. Households pay a large share of THE in Iran as OOP at the point of service delivery. While the household share was more than 50% before the HTP implementation, it was reduced to about 40% after it [19]. NHA categorizes OOP as the main mechanism financed by households in the private sector. The remained share of the private sector is related to the other private agencies [25]. The main features of health financing agents in Iran are presented in Table 1 [19, 26].

**Table1:** Main features of health financing agents in Iran

Financial agencies (Financing schemes)	Proportion of THE (approximate estimation %) from NHA report 2014 [19]	Population size (estimated) [26]	Payment contribution and user charges [26]	Brief description of resources [26]
**Public**	*~ 50*	Total population of country		Includes government and non-government resources
Government	*~ 25*	Total population of country		Includes natural resources (e.g. oil), taxes and other government resources
MSUs	~ 20	Total population of country	Public health and medical services/programs	Public budget allocated by government at provincial and district levels
Others	~ 5	Employees and their dependents mentioned in (~ 5,000,000)	Medical services/programs	Including Municipalities, Armed Forces Medical Insurance, Medical Organization of Petroleum Industry, National Broadcasting Organization, etc
Non-government	*~ 25*		Mainly health insurance schemes	Includes basic and supplementary health services insurances and other non-government resources
HIO	~ 10	Rural Residents	Payment contribution: 6% of the minimum wage (paid by the government) User charges: 5 and 30% of in-patient and out-patients health services, respectively, based on medical tariffs in public sector. Also the gap between private and public tariffs in private centers	Basic social health insurance which is financed by premiums paid by employee and employer and public subsidies
		Government Employees Iranians Fund	Payment contribution: 6% up to twofold of the minimum wage (paid by employee, employer, and government and payment contribution for Iranians Fund and Other Sectors are fixed premiums (50% by the government)	
		Other Sectors (34,123,681)	Fixed premiums (paid by the government) User charges: 10 and 30% of in-patient and out-patients health services, respectively, based on medical tariffs in public sector. Also the gap between private and public tariffs in private centers	
SSIO	~ 13	Employees of the formal private sectors, self-employed and voluntary contributors (37,547,508)	Payment contribution: 30% (20% employers, 7% workers, 3% government) of which 9% is for health benefit package User charges: 10% and 30% of in-patient and out-patients health services, respectively, based on medical tariffs in public sector. Also the gap between private and public tariffs in private centers. No copayment in SSIO’s hospitals and health centers	Basic social health insurance which is financed by premiums of paid by employee and employer
Others	~ 2	2,146,571 and uninsured people	Payment contribution: 6% of the minimum wage (paid by the government) 13% of wage without ceiling for Iran Insurance Company and Central Insurance Company (4% employee and 9% employer) User charges for Imam Khomeini Relief Foundation: Free of charge for inpatient health services in public sector, and 30% of out-patients services. A part of the gap between private and public tariffs in private centers is covered by the this scheme Other medical insurance funds are free of charge in both private and public health centers	Imam Khomeini Relief Foundation, Red Crescent Society, etc
**Private**	*~ 50*		Mainly user fees paid by people and households at the point of service delivery	Includes OOPs paid by Households and other private and charity organizations
OOP	~ 40		Mainly user fees paid by people and households at the point of service delivery	
Others	~ 10		Providers’ investment in private sector for health service delivery	Non-profit institutions serving households, Private Supplementary Insurance Companies, Banks, etc
THE	100 (823,000 billion rials)			Includes total current health expenditures (TCHE) and governance, and health system and financing administration except for some resources such as education and research

## Materials and methods

This descriptive study was conducted using data obtained from national and provincial health accounts in Iran from 2008 to 2016. The main indicators of provincial health accounts include THE, public health expenditures, private health expenditures, OOP, HIO, SSIO, and funding of MoH and MSUs in different provinces of the country. The unit of all monetary amounts was reported per 1000 Rials (Iran’s currency).

The first level is THE, which is formed on the second level, through the algebraic addition of public and private health expenditures. In the third level, the most important subsectors of public health expenditures include allocations to the MoH, MSUs, SSIO, and HIO. The most contribution of the private health care expenditures includes the OOP directly paid by the households at the point of receiving health care services [15, 16, 27].

The GC was employed to measure inequality. This index ranges from zero to one, indicating zero and maximum inequality, respectively [17]. A GC of 0.2–0.35 is considered to be a relatively fair distribution, 0.35–0.5, a relatively unequal distribution, 0.5–0.6, a highly unequal distribution, and > 0.6, a very highly unequal distribution. The GC is calculated using the following formula [17]:

$$G=1-\sum_{k=1}^{n}\left({X}_{k}-{X}_{k-1}\right) ({Y}_{k}+{Y}_{k-1})$$

where *G* is the GC, *X*_*k*_ is the same cumulative share of population variable, *Y*_*k*_ is the cumulative share of each variable associated with financing agents in the present study, and *k* is the study unit, which is the number of provinces. To calculate the GC, the cumulative percentage of the indices was located on the vertical axis, and the cumulative percentage of the country provinces was located on the horizontal axis. In this study, the GC was calculated based on the population and the number of health service providers in each province. In other words, the horizontal axis was once taken to represent the—population variable cumulative share in the province and once to represent the cumulative share of health service providers in each province and was separately calculated for each GC for each index related to financing agents.

To estimate the dispersion rate, the following formula was used [28]: CV = σ/μ, where standard deviation (σ) is divided by the mean of the desirable variable (μ), which means how data are scattered around the mean as a standard. In this study, this indicator was used to indicate the dispersion of each indicator related to financing agents compared to its average. In addition, the Rate Ratio (RR) was employed to determine the difference between the highest and lowest value of each one of studied NHA indicators across the provinces of Iran. In order to better comparison CV and RR measures among the provinces of Iran, we calculated all the studies indicators per capita annually (values divided by population of each province).

We also used the disparity index to indicate the dispersion rate of the OOP contribution in each province to the 30% target in NDPs [22, 23]. This index is used to show the geographic disparities with the targeted or desirable value. In other words, this index measures the difference in the value of an indicator in each region/province, or population group with the targeted or desirable value (a standard level in the country level). This index is calculated using the following formula [29]:

$$ID=\left\{\frac{\frac{\sum \left|{O}_{ri}- {O}_{rl}\right|}{I}}{{O}_{rl}}\right\}\times 100$$

where *O*_*ri*_ is the percentage of the OOP of the studied provinces for the studied years, *O*_*rl*_ is the percentage of targeted OOP declared in NDPs, which is equal to 30%, and *l* is the number of provinces with an OOP higher than 30%. It should be noted that the provinces whose OOP was lower than 30% were excluded from the total number of provinces since they had already achieved the desired target. Data were analyzed using Stata version 14.0.

## Results

Tehran province, the largest province by population, has spent several times more resources than the smallest one.

The obtained results are described in three sections: descriptive, GC, and dispersion of indicators. Findings related to the GC were calculated in two forms of the population base and the number of service providers in each province.

### GC based on province population

The GC of THE increased from 0.51 to 0.54 over the studied years, indicating a high level of inequality in health financing distribution. The inequality in health financing distribution for the private sector also increased from 0.52 to 0.59 during the studied years. Moreover, OOP, as a subset of private expenditures, had a high unequal, and its highest level was 0.61 in 2015. The distribution of public expenditures from 2008 to 2012 was moderately unequal, dropping from 0.5 to 0.48. Then, this value turned into extremely unequal from 2013 to 2015, between 0.51 and 0.52, and falling again to 0.48 in 2016. In case of agents covered by public sector, the results showed that MSUs expenditures had relatively fair distribution ranging 0.33–0.44 during the studied years. Moreover, HIO expenditures showed a relatively unequal distribution of financial resources ranging from 0.39 in 2009 to 0.43 in 2013. Furthermore, in case of SSIO expenditures, the findings showed that there is a high inequality in health financing distribution varied from 0.48 to 0.57 during the studies years (See Table 2).

**Table 2:** Gini coefficient based on the population base for selected indicators of national health accounts from 2008 to 2016

Year	2008	2009	2010	2011	2012	2013	2014	2015	2016
THE	0.51 (0.42–0.60)	0.52 (0.44–0.60)	0.49 (0.41–0.58)	0.50 (0.42–0.58)	0.52 (0.43–0.60)	0.53 (0.44–0.62)	0.53 (0.45–0.62)	0.48 (0.41–0.56)	0.54 (0.46–0.62)
* Public*	0.50 (0.40–0.59)	0.48 (0.39–0.58)	0.49 (0.39–0.58)	0.48 (0.39–0.58)	0.48 (0.38–0.58)	0.52 (0.43–0.61)	0.51 (0.41–0.60)	0.52 (0.44–0.67)	0.48 (0.38–0.57)
* MSU*	0.33 (0.25–0.41)	0.33 (0.25–0.1)	0.33 (0.25–0.41)	0.32 (0.25–0.40)	0.32 (0.25–0.40)	0.44 (0.34–0.55)	0.31 (0.24–0.37)	0.41 (0.35–0.47)	0.34 (0.25–0.43)
* HIO*	0.42 (0.34–0.50)	0.39 (0.32–0.47)	0.40 (0.33–0.47)	0.41 (0.34–0.48)	0.41 (0.34–0.48)	0.43 (0.35–0.50)	0.41 (0.34–0.48)	0.41 (0.34–0.48)	0.40 (0.33–0.47)
* SSIO*	0.55 (0.48–0.63)	0.55 (0.47–0.62)	0.55 (0.47–0.63)	0.56 (0.48–0.63)	0.54 (0.46–0.62)	0.54 (0.46–0.62)	0.54 (0.46–0.62)	0.57 (0.50–0.65)	0.48 (0.39–0.56)
* Private*	0.52 (0.43–0.61)	0.55 (0.48–0.63)	0.50 (0.41–0.59)	0.53 (0.45–0.60)	0.55 (0.48–0.62)	0.53 (0.45–0.62)	0.56 (0.49–0.64)	0.52 (0.37–0.67)	0.59 (0.53–0.66)
* OOP*	0.5 (0.41–0.60)	0.54 (0.46–0.62)	0.47 (0.38–0.57)	0.49 (0.42–0.57)	0.53 (0.45–0.61)	0.51 (0.42–0.61)	0.55 (0.47–0.63)	0.61 (0.39–0.82)	0.59 (0.51–0.66)

**Fig. 1:**
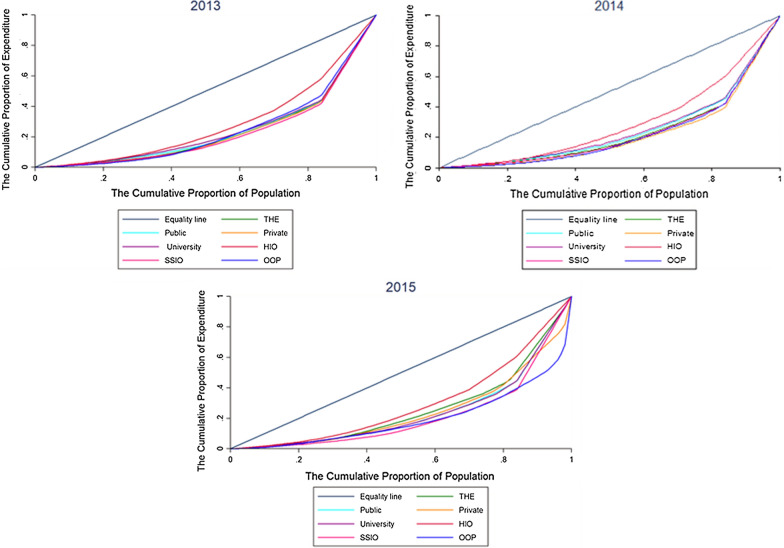
Lorenz curves based on the population base for selected indicators of national health accounts in the years before and after implementing the health transformation plan of Iran

Lorenz curves display the inequality of financing agents’ indicators based on the population of each province for the years before and after the HTP implementation (from 2013 to 2015) (See Fig. 1).

### GC based on the number of service providers

In case of measuring the inequality by number of service providers, THE showed an irregular trend ranging from 0.46 to 0.51 during the studied years which indicates a relatively unequal in health financing distribution. About the private expenditures, there was a high inequality in distribution of financial resources varied from 0.47 to 0.54 during the period. The OOP spent by households ranged from 0.44 to 0.58 with a high inequality coefficient. The distribution of financial resources in the public expenditures was relatively unequal and varied from 0.46 to 0.50 over the years studied. In case of agents covered by public sector, both the MSUs and HIO expenditures showed a relatively unequal, while SSIO showed a high inequality in distribution of financial resources (See Table 3).

**Table 3:** Gini coefficient is based on the number of service providers for selected indicators of national health accounts from 2008 to 2016

Year	2008	2009	2010	2011	2012	2013	2014	2015	2016*
THE	0.48 (0.36–0.60)	0.50 (0.40–0.60)	0.47 (0.37–0.57)	0.48 (0.39–0.56)	0.50 (0.43–0.57)	0.50 (0.41–0.60)	0.51 (0.40–0.61)	0.46 (0.38–0.55)	–
* Public*	0.47 (0.34–0.60)	0.46 (0.34–0.58)	0.47 (0.36–0.58)	0.46 (0.37–0.56)	0.47 (0.39–0.54)	0.50 (0.41–0.59)	0.48 (0.38–0.59)	0.50 (0.41–0.59)	–
* MSU*	0.49 (0.38–0.61)	0.53 (0.44–0.62)	0.47 (0.37–0.57)	0.50 (0.42–0.57)	0.53 (0.46–0.60)	0.51 (0.42–0.60)	0.54 (0.45–0.63)	0.50 (0.35–0.65)	–
* HIO*	0.31 (0.22–0.41)	0.32 (0.23–0.41)	0.32 (0.23–0.41)	0.31 (0.23–0.39)	0.31 (0.24–0.39)	0.43 (0.34–0.53)	0.29 (0.22–0.37)	0.39 (0.32–0.46)	–
* SSIO*	0.40 (0.30–0.49)	0.38 (0.29–0.46)	0.38 (0.30–0.46)	0.39 (0.31–0.47)	0.39 (0.32–0.47)	0.41 (0.33–0.49)	0.39 (0.31–0.47)	0.39 (0.31–0.46)	–
* Private*	0.52 (0.41–0.63)	0.52 (0.42–0.62)	0.52 (0.43–0.61)	0.53 (0.45–0.61)	0.52 (0.45–0.59)	0.52 (0.44–0.60)	0.51 (0.41–0.61)	0.54 (0.45–0.63)	–
* OOP*	0.48 (0.36–0.59)	0.52 (0.43–0.61)	0.44 (0.34–0.55)	0.46 (0.37–0.55)	0.50 (0.42–0.58)	0.48 (0.39–0.58)	0.52 (0.43–0.61)	0.58 (0.34–0.81)	–

*Data on the number of service providers in 2016 were not available

**Fig. 2:**
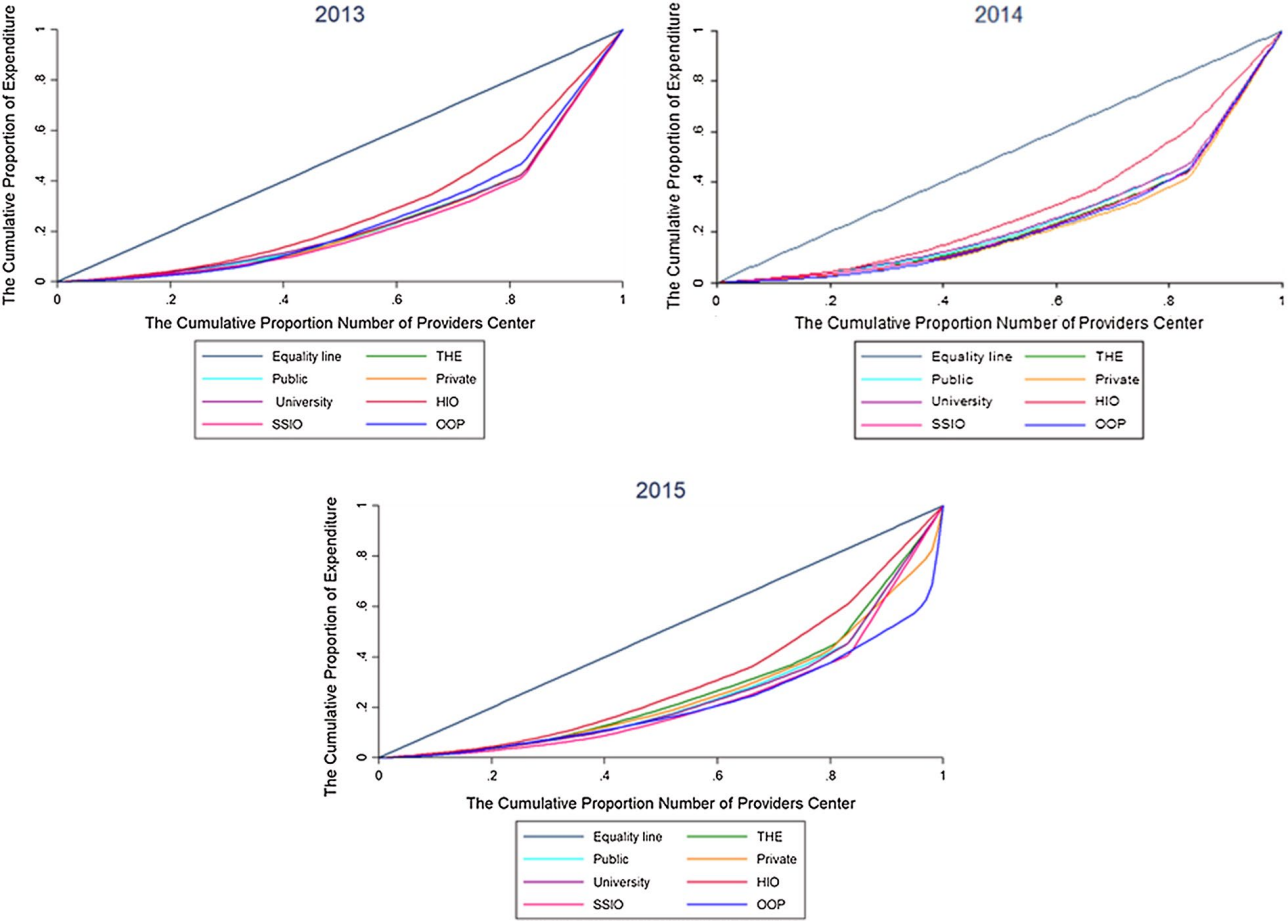
The Lorenz curves based on the number of service providers for selected indicators of national health accounts in the years before and after implementing the health transformation plan of Iran

Figure 2 illustrates the Lorenz curves based on the number of service providers in each province for the years before and after the HTP implementation (from 2013 to 2015).

### Disparity and difference indicators of financial agents

Table 4 shows THE per capita, CV, and RR for the financing agents during the studied years. As presented in the table, there is disparity and remarkable difference in financial resources across the provinces. The results related to the distribution of financial resources based on the population showed a remarkable disparity and difference for THE, public, and private expenditures indicators from 2008 to 2016 as similar with the GC results. Furthermore, the trend of results obtained from the CV and RR showed that rate of difference and variation across the provinces following the HTP introduction in 2014 decreased and then this trend did not persist, and showed an increase again for years 2015 and 2016. Most difference among the provinces was related to private expenditures especially OOP spent by households.

**Table 4:** Coefficient of variation and the rate ratio for selected indicators of national health accounts from 2008 to 2016

Year	2008	2009	2010	2011	2012	2013	2014	2015	2016
THE									
THE Per Capita*	2610.80	3288.76	3977.47	4367.83	5329.24	7661.40	10,646.18	12,573.36	16,195.30
CV	0.27	0.27	0.27	0.25	0.28	0.25	0.22	0.39	0.24
RR	3.84	4.04	4.61	2.56	2.99	3.35	3.32	3.04	3.33
*Public*									
Public Per Capita*	1063.88	1421.54	1571.90	2021.23	2210.29	2997.61	5247.42	5989.724	7691.95
CV	0.18	0.17	0.17	0.18	0.18	0.19	0.18	0.23	0.21
RR	1.98	1.96	2.03	2.11	2.38	2.38	2.34	3.12	3.04
*MSU*									
Private Per Capita*	1545.32	1867.05	2405.40	2346.39	3118.67	4663.51	5398.46	6582.18	14,449.36
CV	0.41	0.42	0.42	0.42	0.45	0.34	0.36	0.43	0.42
RR	14.26	16.52	16.98	5.80	6.12	4.86	6.89	6.52	7.25
*HIO*									
University Per Capita*	397.06	18,128.23	17,977.87	26,192.53	24,520.46	33,301.44	65,778.43	2227.97	77,721.84
CV	0.25	0.25	0.25	0.30	0.27	0.24	0.23	0.26	0.34
RR	4.39	4.55	4.74	4.63	4.47	3.45	2.91	3.32	5.19
*SSIO*									
HIO Per Capita*	151.88	244.68	310.97	361.23	446.93	585.21	1096.96	1565.27	1859.47
CV	0.20	0.18	0.18	0.18	0.19	0.19	0.19	0.19	0.19
RR	2.81	2.73	2.62	3.15	3.45	3.18	3.46	3.12	2.87
*Public*									
SSIO Per Capita*	303.83	383.22	438.67	516.17	667.17	940.68	1451.52	1466.93	2485.36
CV	0.40	0.38	0.37	0.39	0.38	0.37	0.36	0.41	0.45
RR	4.50	4.84	4.58	5.03	5.56	5.36	5.04	5.31	5.45
*OOP*									
OOP Per Capita*	1330.94	1599.32	2030.02	1835.09	2406.74	3793.73	4382.94	5293.21	6383.73
CV	0.45	0.46	0.48	0.50	0.54	0.38	0.42	0.49	0.49
RR	6.62	14.18	9.67	11.89	13.84	6.74	9.73	9.84	9.98

*The values are reported in thousand Rails (Iranian currency).

**Fig. 3:**
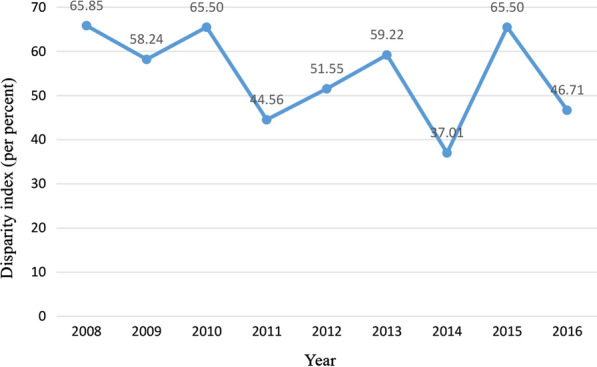
Geographic disparities of OOP proportion from 2008 to 2016 in Iranian provinces using disparity index

The results of disparity index for OOP showed that the values of this index from 2008 to 2016 has a fluctuating and irregular trend. Regional disparities in OOP proportion and were varied from 37.01 to 65.85% during the studied years. In general, during the first year of the HTP implementation, the disparity value of OOP across the provinces decreased from 59.22 to 37.01%, indicating a 37.5% reduction in regional disparity and then increased by 76.90 and 26.20% in 2015 and 2016, respectively [See Fig. 3].

## Discussion

NHA provides a framework to collect, compile, and analyze such data on all types of health spending in a country. As, institutionalization of NHA provide a strong evidence base for decision making in order to creating new resources, reallocating existing resources, improving efficiency of current spending and improving the equity in health financing. International reports show that developing countries such as Turkey, Philippines and Malaysia regularly use NHA framework to enable the government to identify health system issues and rearrange the policies accordingly. NHA also can help to successful implementation, evaluation, and management of health reform [8]. Meanwhile, one of policy implications of NHA is how the indicators are varied across different provinces within a country, which aspires equity in health financing as the main concern worldwide [30], and inequity in health expenditures is one of the factors affecting the health of households. Therefore, this study aimed to investigate the inequality in the indices of the health care system financing agents from 2008 to 2016, based on population and the number of service providers.

Overall, we found that there is inequality in the resources allocated by financing agents studied in Iran. Moreover, the private sector particular the subset of OOP had the highest inequality among main financing agents. In case of public expenditures, we also delineated that highest inequality in resources distribution is related to SSIO. Surprisingly, the inequality measured by the number of service providers in each province was less than the inequality per the population base. The results were discussed in detail as follows:

According to the results, the distribution of THE was relatively unequal or moderately unequal, and its distribution process was more or less incremental in terms of the population, which inequality was higher than inequality in terms of the number of health care providers. More precisely, inequality in the distribution of resources in terms of population is greater than equality based on the number of health service providers. One of the main reasons seems to be this issue that allocating funds to the provinces by the main agents are relied on the structure of service provision. However, several other studies demonstrated that health allocation patterns based on the geographical conditions, population, disease burden, and the need in each region are better than the health allocation based on the number of health care providers. The results of a study on the allocation of resources to the health system in Iran show that the basis for allocating more resources is based on the defined service delivery structures and that consequently, demographic and epidemiological indices in this model are deemphasized, which is consistent with the results of this study [31, 32]. As a result, it is suggested that policymakers should pay more attention to the variables associated with demographic, epidemiological, and geographical conditions in the allocation of health system resources.

The results also show that inequality in the distribution of private health expenditures was higher than inequality in the distribution of public health expenditures, and its trend showed a sharper increase. Hence, the pattern of inequality was similar in both bases of GC (i.e., population and the number of health service providers) in terms of the main agents of financing, including MSUs, HIO, and SSIO. This issue can be due to at least three reasons. Firstly, the distribution of disease burden varies from one region to another region, and this is more important, especially for chronic and non-contagious diseases, which have higher expenditures. Therefore, various studies conducted in Iran indicate that inequality in risky behaviors, access to health services, and health outcomes such as mortality and morbidity, are evident in urban, rural areas and different provinces [33–36]. Secondly, as the main incentive of the private sector in delivering health services is the attained profit, regions with more facilities, higher development level, and stronger infrastructures provide more potential opportunities for private sector development. Thus, these sectors focus on further investment in these provinces. As a result, regarding the higher tariffs of the private sector and the lack of appropriate insurance coverage in the private sector, this could increase OOP spending compared to other areas.

Evidence suggests that the services provided by the private sector depend on the mechanism and type of services covered, e.g., whether they are specialized or general, or therapeutic and preventive. Furthermore, the method of payment can affect the access of people to need-based services. Even in most cases, due to the incentives for profitability, financial access is limited, especially for the underprivileged and less developed regions [37], which requires targeted policy and planning, focusing on strategic purchasing of health services by governments to ensure quality and affordability. Thirdly, there are some inequalities in the distribution of health facilities and human resources that can lead to some shortcomings in access to these services in some areas. Consequently, people go to other provinces to receive services, and this increases OOP spending in the destination provinces. Some studies conducted in Iran delineated inequality in the distribution of some health resources and facilities among the country’s provinces [38, 39]. It appears that HTP interventions have not been effective in the balanced distribution of HR in different provinces and cities of the country.

Considering the years before and after the HTP implementation, the results of our study showed that following the beginning of the plan in early 2014, the government allocated significant resources from the targeted subsidies and part of the increase in the value-added tax to the health sector. Nevertheless, it seems that due to adding these new resources, the inequality in the distribution of public health decreased compared to the year before its implementation. However, inequality in the distribution of private sector resources increased after the implementation of this plan. In the later years of HTP implementation, due to the unmet financial resources by government for this plan, a relative increase in the inequality in public and private expenditures was observed, which was also consistent with other studies [21, 40]. This increase seems to be due to interventions incorporated in HTP were mainly focused on health care providers affiliated with MSUs, and other public sectors such as SSIO, medical centers affiliated with armed forces private sector were excluded. We also concluded that the both distribution of resources based on the population base and the number of health services providers by MSUs has less inequality than basic health insurers (i.e. HIO and SSIO).

Among the main agents of the provision of basic health insurance, HIO had lower inequality in the distribution of resources compared to the SSIO. The probable cause of this difference seems to be that the SSIO, in addition to indirect treatment, provides health care services through direct treatment focused on its customers. Due to the unequal population with health insurance coverage, the distribution of direct treatment centers in provinces of Iran is unequal, and most centers providing direct health care are located in the center of industrial provinces. Therefore, this misallocation may be a reason for inequality in the resource distribution of this insurance organization compared to that of HIO in provinces. Therefore, health insurance policy integration as well as the use of strategic purchasing in basic health insurance, based on the demographic needs of each region, can improve the equity in the distribution of health insurance resources at the provincial level.

The results of the disparity index from the OOP health expenditures showed that during the studied years, the disparity rate from the target index of at least 30% of the OOP according to the fourth and fifth NDPs did not have a regular trend. In conclusion, the disparity of the OOP was decreased after the first year of the HTP implementation as compared with the previous years. This relative increase in the second and third years showed that in the subsequent years, due to the lack of resource allocation to continue the plan and instability of government funding, it led to further disparity and, consequently, distancing from the target of the fifth NDP. Furthermore, the results obtained by Homaie Rad et al. (2017) on the urban family physician program indicated that the implementation of this program not only did not change the OOP compared to the previous years but also increased inequality in the OOP payments [41]. This is due to the incomplete implementation of the family physician program and its related components as well as severe financial constraints in the aftermath of economic instability. It should be noted that necessarily applying economic reforms cannot improve the equity in health financing, such as what was concluded in a study concluded in Iran indicating targeted subsidies law could not improve the equity in health financing [42].

### Study strength and limitations

This study is one of the first studies investigating the inequality in the distribution of health resources in terms of financial resource agents for almost a decade at the provincial level in Iran. Although this study was based on the NHA indicators, some indicators such as donors support, non-profit institutions serving households, banks, and other insurance organizations, including the armed forces and private companies, were not included. However, these indicators consist of approximately 15% of THE [25, 43]. Thus, the indicators with a greater share of funding, including SSIO, HIO, MSUs, and OOP spent by households, were included in our study. Another limitation of the present study is the lack of access to data on the number of service providers in 2016; therefore, the GC was calculated based on the number of service providers for health financing agents from 2008 to 2015.

## Conclusion

NHA provide a framework to collect, compile, and analyze such data on all types of health spending in a country—and so create a robust evidence base for policymaking. Inequality in the distribution of health financial resources can affect health outcomes. According to the results, inequality in the main indicators of health financing agents, especially public sector, including MSUs, HIO, and SSIO, was moderate to high. Moreover, inequality in private health expenditures was higher than public one. In the sector related to public health expenditures, the SSIO agent had a higher level of inequality compared to the HIO and MSUs. Moreover, inequality in the distribution of private sector increased after the HTP implementation. It seems that unmet resources by government for this plan, increased the inequality in health resources. Therefore, to distribute equitable financial resources, some policy recommendations are suggested. First, the distribution of resources and human resources based on HTP interventions should be readdressed. Second, policy integration strategy of health insurance funds based on the principles of strategic purchasing and quality of services tailored to the needs of the population should be considered. Third, the implementation of a family physician program in the whole country based on an interactive referral system between service delivery levels and continuity of information through institutionalizing the electronic health record of the households should also be readdressed. Increasing share of health financing agents, including government and basic social health insurers, in provincial distribution based on the epidemiological, demographic, and geographical situations needs to be taking in to account. Last but not least, the GC, disparity, and CV are useful measures to illustrate how resources are distributed at the geographical level, and it is highly suggested that these measures be considered in NDPs.

## Abbreviations

GC: Gini Coefficient
CV: Coefficient of Variation
RR: Rate Ratio
OOP: Out-Of-Pocket
HIO: Health Insurance Organization
HTP: Health Transformation Plan
THE: Total Health Expenditure
NDP: National Development Plan
CHE: Catastrophic Healthcare Expenditure
MoH: Ministry of Health
MSUs: Medical Sciences Universities
SSIO: Social Security Insurance Organization
AFMSIO: Armed Forces Medical Services Insurance Organization
IKRF: Imam Khomeini Relief Foundation
NHA: National Health Accounts
